# Prediabetes is associated with a higher serum neurofilament light chain level in adolescents

**DOI:** 10.3389/fendo.2023.1207045

**Published:** 2023-06-26

**Authors:** Zheng Chen, Lan-Ping Wu, Tuo-Chao Peng

**Affiliations:** Department of Anesthesiology, Hunan Children’s Hospital, Changsha, China

**Keywords:** prediabetes, adolescents, sNfL, cognitive dysfunction, retrospective cohort study

## Abstract

**Objective:**

Serum neurofilament light chain (sNfL) level, which is a biomarker indicative of neuroaxonal damage and cognitive impairment, has been reported in several neurological diseases. There has been a lack of studies on the association between sNfL levels and prediabetes in adolescents. This study investigated whether sNfL levels were higher in adolescents with prediabetes undergoing elective orthopedic surgery.

**Methods:**

The sNfL level was measured in 149 adolescents aged from 12 to 18 years who underwent elective orthopedic surgery at the Hunan Children’s Hospital (18 with and 131 without prediabetes). We evaluated the association between prediabetes and sNfL level after adjusting for age, sex, and triglycerides using a multivariable linear regression model.

**Results:**

The prevalence of prediabetes in adolescents was 12.08%. Univariate logistic regression analysis showed that prediabetes was related to sNfL. In multivariate logistic regression analysis, the association between prediabetes with sNfL levels remained significant after adjustment for age, sex, and triglyceride. The relationship between the two was further visualized by a smoothed curve.

**Conclusions:**

Prediabetes is associated with a higher sNfL. Further large-scale and prospective studies are needed to verify the clinical application of sNfL as a monitoring biomarker for adolescent prediabetes in adolescents and to evaluate the performance of sNfL in predicting the incidence of neuropathy and cognitive dysfunction in adolescents with prediabetes.

## Background

Due to increasing obesity, diseases previously almost exclusively found in adults, such as prediabetes and type-II diabetes(T2D), are now frequently diagnosed among adolescents (1). Prediabetes is a term that generally refers to an intermediate state of abnormal glycemia. In the United States, the National Health and Nutrition Examination Surveys have shown that the prevalence of prediabetes among adolescents is approximately 18.0% (2). Meanwhile, prediabetes is a pressing clinical and public health issue, as studies have reported that the highest risk of diabetes, major adverse cardiovascular events (MACE), and chronic kidney disease occur in individuals with prediabetes (3, 4). As a precursor of T2D, prediabetes is more severe in adolescents than in adults because the accelerated period of progression from prediabetes to T2D (5, 6) could lead to an early onset of complications and adverse events that affect patients’ quality of life and long-term outcomes. The increasing prevalence of prediabetes in adolescents has now become one of the major public health concerns worldwide (7).

Neurofilaments are considered major cytoskeletal components of neurons and are classified into light, medium, and heavy chains according to the size of the proteins. Neurofilament proteins are enriched in axons, simultaneously providing mechanical support and maintaining axon homeostasis (8, 9). Neurofilaments can be released from damaged or diseased axons in significant amounts into the blood and cerebrospinal fluid (CSF), and therefore their elevated levels are often used as potential biomarkers to indicate various neurological diseases (10, 11). CSF is the most frequently used biofluid for measuring neurofilament light chain (NfL) levels in neurodegenerative diseases, but because of the invasiveness of lumbar puncture as well as the pain and distress associated with CSF collection, it is impractical to obtain CSF from adolescents, who require strict instruction (12). The application of novel highly sensitive analytical methods has made it possible to measure low levels of NfL in blood with high accuracy and reproducibility. As several studies have demonstrated close correlations between CSF and sNfL (13, 14), it is practical to study sNfL in a wide range of neurological disorders. However, to properly explain sNfL levels in relation to disease states, it is also important to consider factors relevant to this protein change. Recent studies have shown that sNfL may be affected by some factors such as age, systolic blood pressure (SBP), and body mass index (BMI) (15–17).

Traditionally, neuropathy has been considered a microvascular complication that occurs in patients with a long history of diabetes. More recently, however, it has been reported that neuropathic complications may develop as early as the time of diagnosis of diabetes mellitus (18–20). The prevalence of documented neuropathy in individuals with prediabetes is approximately 11%-25% (18). Prediabetes has been increasingly recognized as an important factor leading to neuropathy. In particular, emerging data and epidemiologic studies support that prediabetes is a risk factor for mild cognitive impairment (21–23).

There is a lack of studies on prediabetes in adolescents. This study was designed to identify the main contributors to the sNfL level in perioperative adolescents and to assess whether a higher sNfL level was related to adolescents with prediabetes in elective orthopedic surgery. The current data analyzed in our study were obtained from Hunan Children’s Hospital.

## Methods

The data for this study were collected from the electronic medical records (2021-2022) of the Hunan Children’s Hospital. This retrospective study was approved by the Ethics Committee of Hunan Children’s Hospital [No. HCHLL-20230-43]. Informed consent was waived due to the observational nature of the study.

This study contained anonymous demographic, medical, surgical, and laboratory information from adolescents undergoing elective orthopedic surgery in the Department of Anesthesiology. All adolescent patients (aged from 12 to 18 years old) had a random preoperative plasma glucose or hemoglobin A1c. Exclusion criteria were patients undergoing emergency surgery, age <12 years old, ASA grade VI, no sNfL data, or no plasma glucose. The present analysis was exempted due to the deidentified dataset of the study. The study was conducted following a pre-specified protocol and statistical plan that was not disclosed prior to data analysis. This manuscript was prepared according to the Strengthening the Reporting of Observational Studies in Epidemiology (STROBE) guidelines.

### Laboratory tests and clinical data

Patient data were collected. This included age, sex, and body mass index (BMI), calculated as weight in kilograms divided by height in meters squared.

Laboratory methods were used to measure hemoglobin A1c(HbA1c), plasma glucose, high-density lipoprotein (HDL), systolic blood pressure (SBP), sNfL, cholesterol, and triglyceride levels. The single point insulin sensitivity estimator (SPISE) was calculated using the following formula: [600* HDL^0.185/(TG^0.2* BMI^1.338)] (24).

### Definition of prediabetes

Prediabetes was defined in accordance with the American Diabetes Association criteria when any of the following conditions were met: (1) hemoglobin A1c (HbA1c) level≥5.7% and <6.5% (25); and (2) a random plasma glucose≥100 mg/dL and <160mg/dL (26).

### Measurement of sNfL

Frozen serum was thawed on ice and then spun prior to NfL measurement using the Simoa HD-X Analyzer (Quanterix) and Single Molecule Array (Simoa) technology according to the manufacturer’s instructions. The assay for these samples had a lower limit of detection of 0.038 pg/mL, a lower limit of quantitation (LLOQ) of 0.174 pg/mL, a dynamic range of 0–2,000 pg/mL, and a coefficient of variation of 7% at the LLOQ. Measurements were performed in duplicate and lots were completed by an investigator blinded to all clinical data, including outcome measures. Samples with a between-measurement coefficient of variation >20% were repeated according to standard practice.

### Statistical analysis

All the analyses were conducted using IBM SPSS (version 22.0) and R (version 4.12.0) software. Data were presented as median (M) and interquartile range (IQR). The Wilcoxon rank-sum test was used to compare continuous variables with non-normal distributions, and the chi-squared test was used to compare the composition ratio of classified data. The association between prediabetes and sNfL level was modeled using multivariate linear regression analysis. The selection of covariates to be included in the model was based on data from previous studies in which age, sex, prediabetes, and triglycerides were found to be associated with sNfL level. A two-tailed value of P<0.05 was considered statistically significant.

## Results

### Features of the study population

**Figure 1:**
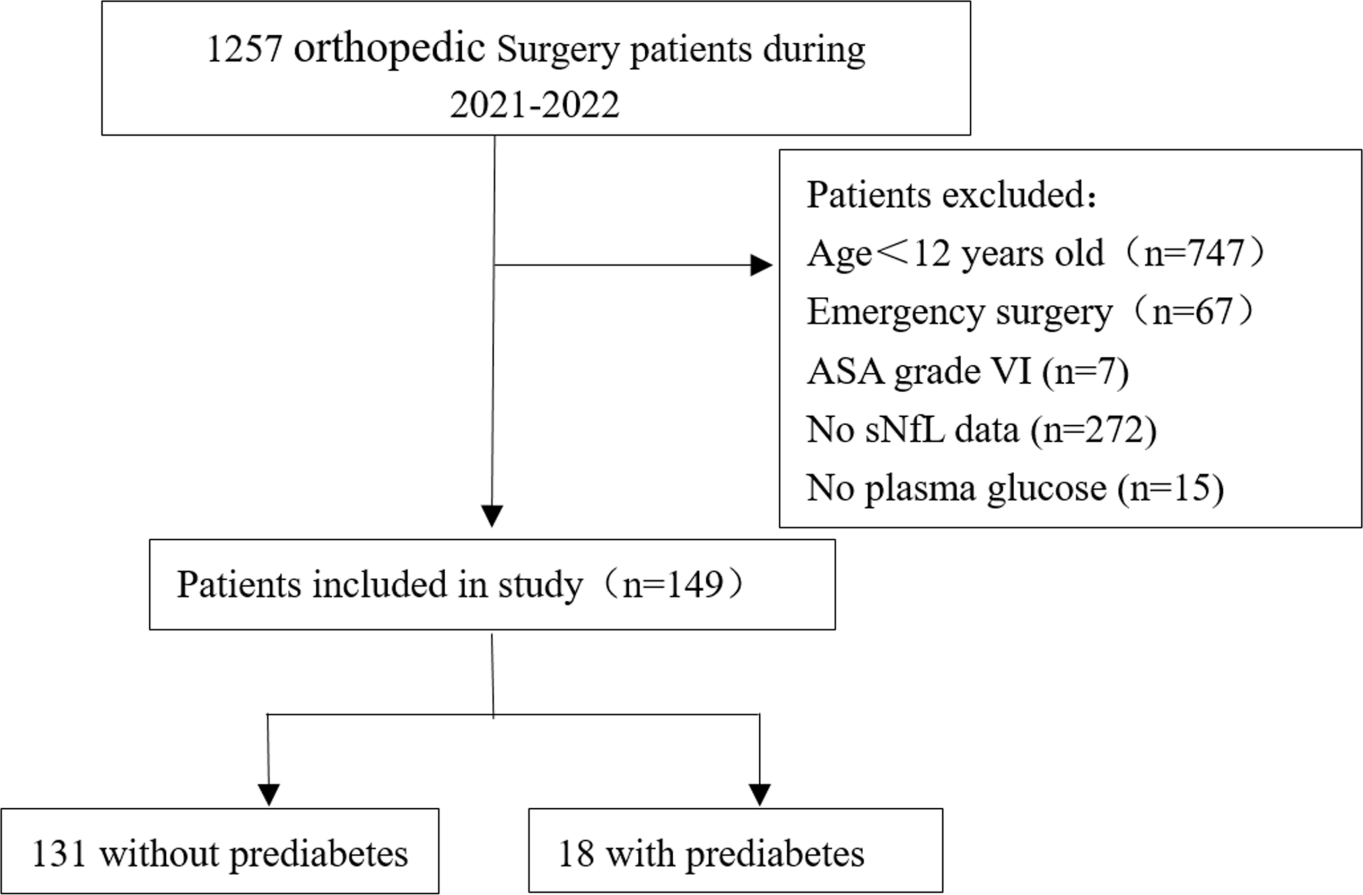
Flowchart of patients’ selection.

The process of patient selection was shown in **Figure 1**. A total of 149 patients were eligible for analysis, including 131 participants without prediabetes and 18 patients with prediabetes (12.08%). All the adolescents were grouped according to whether they had prediabetes before surgery. The overall characteristics of the study population were shown in **Table 1**. The median [IQR] sNfL of adolescents with and without prediabetes before surgery was 13.500 mmol/L and 9.750 mmol/L, respectively, with a statistically significant difference (P=0.029). Other factors including gender, age, BMI, cholesterol, triglycerides, HDL, SBP, and SPISE were not significantly different between the two groups.

**Table 1:** Characteristics of participants according to quartiles of serum neurofilament light chain (sNfL) levels.

Variables	Without prediabetes (N=131)	With prediabetes (N=18)	P-value
Male,%	51.50	48.01	0.511
Age, years	14.636 ± 1.593	14.556 ± 1.854	0.960
sNfL, mmol/L	9.750 (7.275-14.175)	13.500 (9.850-20.775)	0.029*
BMI, kg/m^2^	23.005 ± 1.382	23.260 ± 1.555	0.470
Cholesterol, mmol/L	4.770 ± 0.988	5.106 ± 1.177	0.188
Triglyceride, mmol/L	92.000 (62.000-134.500)	102.000 (72.250-146.750)	0.662
HDL, mmol/L	47.000 (41.000-58.000)	51.500 (40.500-54.500)	0.808
SBP, mmHg	110.500 ± 12.011	112.667 ± 11.003	0.470
SPISE	7.542 ± 1.386	7.460 ± 1.338	0.813

BMI, Body Mass Index; HDL, High-Density Lipoprotein; SBP, Systolic Blood Pressure; SPISE, Single-Point Insulin Sensitivity Estimator.

*: P<0.05.

### Univariable analysis of sNfL

**Figure 2:**
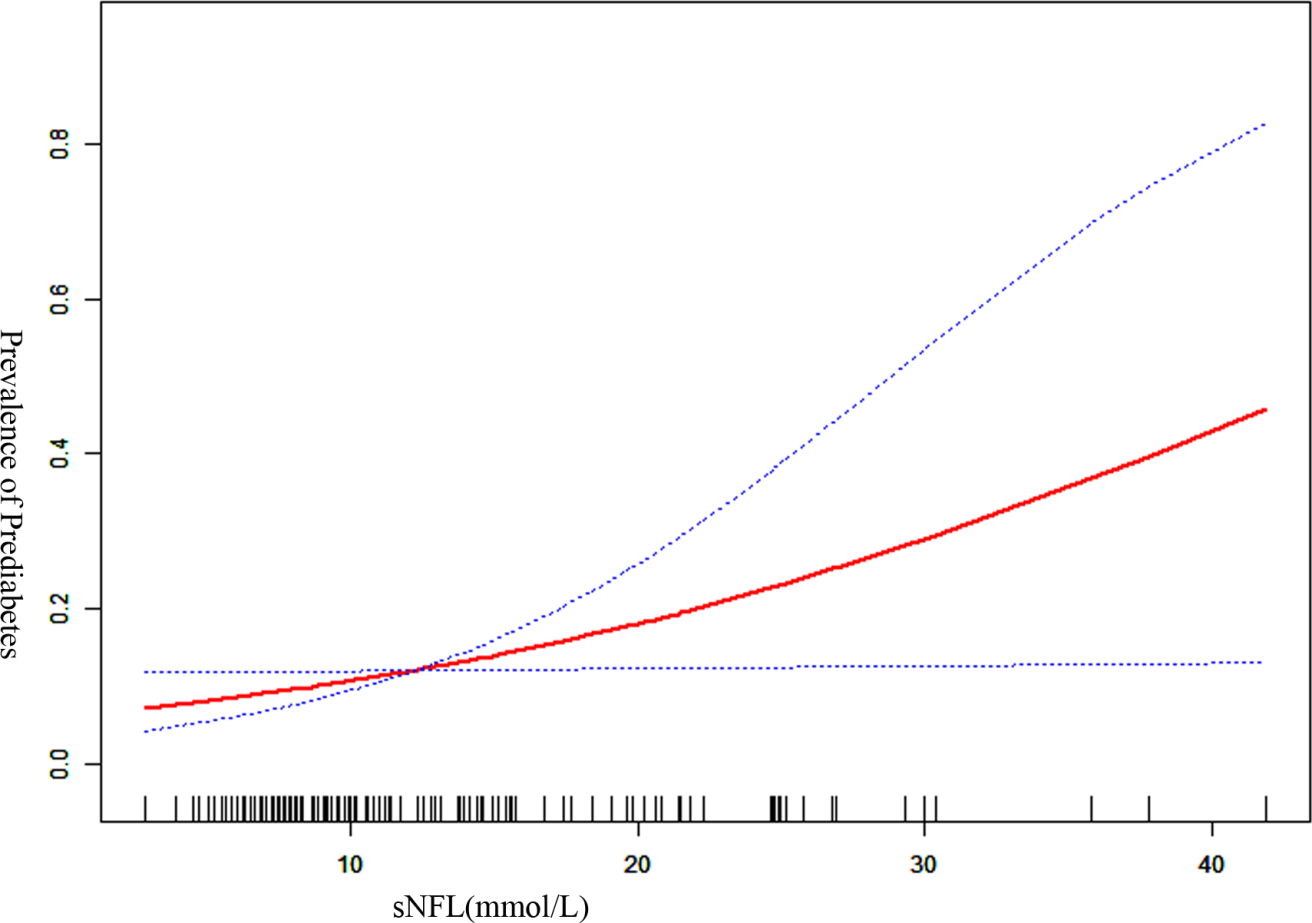
Smoothed curve of the sNfL and Prevalence of Prediabetes.

As shown in **Table 2**, when considering the entire cohort, the univariable analysis showed that prediabetes (RR, 4.147; 95%CI, 0.584-7.710, P=0.024) was associated with sNfL. Apart from prediabetes, other factors, such as gender, age, BMI, cholesterol, triglycerides, HDL, SBP, and SPISE, were irrelevant to sNfL. In **Figure 2**, a smoothed curve was applied to visualize the relationship between sNfL and the prevalence of prediabetes in adolescents, indicating that the prevalence of prediabetes was correlated with sNfL.

**Table 2:** Univariable analysis of sNfL.

Exposure	Statistics	sNfL, RR (95%CI)	P-value
Prediabetes	18 (12.08%)	4.147 (0.584, 7.710)	0.024*
Age	14.427 ± 1.620	0.312 (-0.416, 1.040)	0.402
SPISE	7.532 ± 1.376	-0.240 (-1.099, 0.618)	0.584
BMI	23.035 ± 1.401	-0.158 (-1.001, 0.686)	0.714
Cholesterol	4.810 ± 1.014	-0.664 (-1.825, 0.497)	0.264
Triglyceride	122.320 ± 106.169	0.006 (-0.005, 0.018)	0.256
HDL	49.173 ± 11.957	-0.022 (-0.120, 0.077)	0.670
SBP	110.760 ± 11.881	0.085 (-0.014, 0.184)	0.093
Male	76 (51.01%)	-2.480 (-6.510, 1.550)	0.230

SPISE, Single-Point Insulin Sensitivity Estimator; BMI, Body Mass Index; HDL, High-Density Lipoprotein; SBP, Systolic Blood Pressure.

*: P<0.05.

### The sNfL level as an independent predictor in adolescents taking elective orthopedic surgery

The association between prediabetes and sNfL level was modeled using multivariate linear regression analysis. The results were shown in **Table 3**. Multiple regression analysis revealed that prediabetes (RR, 4.172; 95%CI, 0.602-0.742, P=0.023) was positively correlated with sNfL independent of other factors in all patients.

**Table 3:** Multivariable linear regression model evaluating predictors of serum neurofilament light chain (sNfL) levels in the studied population.

Variables	RR	95%CI	P-value
(Intercept)	7.222	-7.831, 13.851	0.184
Age	0.269	-0.465, 1.004	0.473
Prediabetes	4.172	0.602, 0.742	0.023*
Male	-2.222	-6.222, 1.779	0.278
Triglyceride	0.006	-0.005, 0.017	0.304

*:P<0.05.

### Sensitivity analysis for sNfL

As shown in **Table 4**, various variables were divided into subgroups, which were subjected to regression analysis to investigate the relationship between the subgroup variables and sNfL using sensitivity analysis. We found that low cholesterol (RR, 10.585; 95%CI, 3.616-17.554, P=0.005), low triglyceride (RR, 13.124; 95%CI, 6.160-20.089, P<0.001), and high HDL (RR, 9.593; 95%CI, 2.964, 16.222, P=0.006) were associated with sNfL.

**Table 4:** Sensitivity Analysis for sNfL.

Subgroups	N	sNfL, RR (95%CI)	P.value
SPISE Tertile			
Low	44	2.230 (-2.823, 7.283)	0.392
Middle	42	5.072 (-3.979, 14.123)	0.279
High	63	4.994 (-0.086, 10.073)	0.059
BMI Tertile			
Low	50	4.408 (-0.772, 9.587)	0.102
Middle	50	4.591 (-2.605, 11.788)	0.217
High	49	4.136 (-2.320, 10.593)	0.215
Cholesterol Tertile			
Low	50	10.585 (3.616, 17.554)	0.005*
Middle	50	5.592 (-4.199, 15.382)	0.268
High	49	-0.285 (-4.287, 3.717)	0.890
Triglyceride Tertile			
Low	50	13.124 (6.160, 20.089)	<0.001*
Middle	50	-0.540 (-6.949, 5.869)	0.870
High	49	0.967 (-4.088, 6.021)	0.709
HDL Tertile			
Low	49	1.324 (-4.439, 7.087)	0.655
Middle	51	2.668 (-3.441, 8.776)	0.396
High	49	9.593 (2.964, 16.222)	0.006*
SBP Tertile			
Low	44	2.230 (-2.823, 7.283)	0.392
Middle	42	5.072 (-3.979, 14.123)	0.279
High	63	4.994 (-0.086, 10.073)	0.059
Gender			
Female	73	3.781 (0.021, 7.541)	0.051
Male	76	8.362 (-2.301, 19.024)	0.150

*:P<0.05.

## Discussion

In the present study, we evaluated the association between a higher sNfL level and prediabetes in adolescents undergoing elective orthopedic surgery. According to the American Diabetes Association criteria, the prevalence of prediabetes in adolescents in our study was 12.08%. The current univariate analysis showed a relation between prediabetes and sNfL. After adjustment for age, gender, and triglyceride, the results showed that the association between prediabetes and sNfL level was still significant. The smoothed curve further visualized the relationship between the two. Our findings demonstrated showed a higher sNfL level in adolescents who had prediabetes, providing neurochemical evidence for subclinical axonal damage and underlying cognitive impairment in prediabetes adolescents. Such a finding could help improve interventions to promote the reversion of prediabetic states to normal glucose tolerance.

Although several studies have examined sNfL levels in patients with various neurological disorders, to the best of our knowledge, this was the first study to report the association of sNfL levels with prediabetes in adolescents. Importantly, the association between prediabetes and sNfL remained significant after adjustment for sex, age, and triglycerides. Previous studies have shown a positive association between sNfL levels and age (27, 28), as a marked increase in sNfL levels has been found to be associated with older age, particularly in those over 60 years of age (29). Our results did not over 60 years of age. Similarly, there was no independent association between sex and sNfL in our study, which was generally over 60 years of age studies (30, 31).

Neurofilaments are released into the extracellular space following neuroaxonal damage, and we found that prediabetes was associated with a higher sNfL. Our study was the first to validate a higher sNfL as a potential biomarker of mild nerve axon damage or milder peripheral neuropathy in prediabetic adolescents. It was well known that neuropathy is one of the major causes of complications in the diabetic population, however, but some studies reported that peripheral neuropathy may develop in humans with prediabetes before overt hyperglycemia (19, 32, 33), suggesting that peripheral nerve injury may occur in the early stages of the disease with milder glycemic dysregulation. In general, prediabetic neuropathy is milder than diabetic neuropathy and primarily affects nerve fibers that mediate sensory function (34, 35). Currently, there is increasing scientific evidence indicating that sNfL is correlated with neurodegenerative diseases (36) and peripheral neuropathies in both humans and animals (37–40). Some studies have observed that diabetes is associated with sNfL (41–43) and that individuals recently diagnosed with diabetes provide new evidence that a higher sNfL is related to diabetic sensorimotor polyneuropathy and peripheral nerve dysfunction (44). Our findings extend previous observations by demonstrating the association between sNfL and prediabetes in adolescents. A recent study showed that the level of sNfL is elevated 6 years before the clinical onset of multiple sclerosis (MS), implying that damage to nerve axons has already begun during the long prodromal period before the diagnosis of MS (45). Similar to this study, we showed that in an intermediate state of abnormal glycemia, that is, prediabetes, started to appear potential or minor nerve axon damage began to appear before the diagnosis of diabetes based on the changes in sNfL.

By correlating sNfL with adolescent prediabetes, our study indirectly demonstrated that there may be an underlying cognitive impairment in adolescent prediabetes. Previous studies have shown that performance in some domains of cognitive function appears to be impaired in patients at early stages of the disease, including prediabetes (46, 47). In addition, prediabetic status or progression to the diabetic phase may promote the reversion of mild cognitive impairment (MCI) to normal cognition (48). Current evidence increasingly supports the use of sNfL level as a biomarker for cognitive dysfunction (49–52). Our study found that measuring sNfL in adolescents with prediabetes could help identify young people at risk of developing cognitive impairment. Therefore, it was necessary to measure sNfL in prediabetic adolescents to provide an early opportunity to reverse the prediabetic state to a normal blood glucose state and prevent more serious cognitive impairment.

### Strengths

Our study had several strengths. It was the first study of the relationship between sNfL and prediabetes in adolescents, and such a selection of the study population allowed analyses of associations without the confounding effect of age-related comorbidities. In addition, sNfL levels were measured with a highly reproducible method, and serum samples from cases and controls were analyzed simultaneously in a blinded and randomized fashion, effectively avoiding potential artifactual differences in sNfL concentrations.

### Limitations

Several limitations should also be acknowledged. First, the retrospective design made it impossible for us to know the predictive value of sNfL, and the retrospective study implies the possibility of missing data. Second, we lacked cognitive function tests and objective measurements of sensorimotor neuropathy, so more studies are needed to thoroughly illustrate the relevant contents. Finally, although several variables were adjusted in the analysis, we cannot completely exclude the possibility of residual confounding.

## Conclusion

In conclusion, our study showed that the association between prediabetes and sNfL was significant even after adjustment for several covariates. A higher serum NFL level was associated with prediabetes in adolescents. It also suggested the possibility of potential neurological damage and cognitive impairment in prediabetic adolescents. Further large-scale and prospective studies are needed to verify the clinical application of sNfL as a monitoring biomarker for prediabetes, and we encourage future studies to evaluate the performance of sNfL in predicting the incidence of neuropathy and cognitive dysfunction in adolescents with prediabetes.

## Data availability statement

The raw data supporting the conclusions of this article will be made available by the authors, without undue reservation.

## Ethics statement

The studies involving human participants were reviewed and approved by Hunan Children’s Hospital. Written informed consent to participate in this study was provided by the participants’ legal guardian/next of kin.

## Author contributions

ZC contributed to the conception and design of the study. T-CP performed data collection and statistical analysis. L-PW collated and interpreted the results and wrote the first draft of the manuscript. All authors contributed to the article and approved the submitted version.
